# Inhibitory effect of low-molecular-weight peptides (0–3 kDa) from *Spirulina platensis* on H_2_O_2_-induced oxidative damage in L02 human liver cells

**DOI:** 10.1186/s40643-021-00388-0

**Published:** 2021-05-07

**Authors:** Jun Ma, Xiankun Zeng, Min Zhou, Le Cheng, Difeng Ren

**Affiliations:** Beijing Key Laboratory of Forest Food Processing and Safety, College of Biological Science and Biotechnology, Beijing Forestry University, Beijing, 100083 People’s Republic of China

**Keywords:** *Spirulina platensis* peptides, Antioxidant activity, H_2_O_2_-induced damage, L02 cell

## Abstract

*Spirulina platensis* protein hydrolysates were prepared by digesting protein extracts with papain, and the hydrolysates were separated into 30, 10, and 3 kDa weights using membrane ultrafiltration. The 0–3 kDa low-molecular-weight *Spirulina* peptides (LMWSPs) proved the highest chemical antioxidant activity by 1,1-diphenyl-2-picrylhydrazyl (DPPH) radical scavenging ability, hydroxyl radical (·OH) scavenging activities and total antioxidant capacity. Cellular antioxidant ability of LMWPs fractions against 2000 μg/mL H_2_O_2_ induced oxidative damage of L02 cells were investigated. The MTT assay results displayed that LMWSPs at different concentrations (0–1000 μg/mL) had proliferation effect on the L02 cells and that treatment of the L02 cells with the 1000 μg/mL LMWSPs (0–3 kDa) significantly prevented H_2_O_2_-induced oxidative damage compared with control cells. Moreover, the 2′,7′-dichlorofluorescein diacetate (DCFH-DA) fluorescent probe assay showed that the levels of ROS and NO were significantly lower in the experimental group that was treated with the peptides for 24 h than in the control group. Furthermore, using the corresponding kits, the treatment inhibited the reduction of SOD activity and the increase of MDA contents in the L02 cells. Therefore, LMWSPs (0–3 kDa) may have potential applications in antioxidant and liver health products.

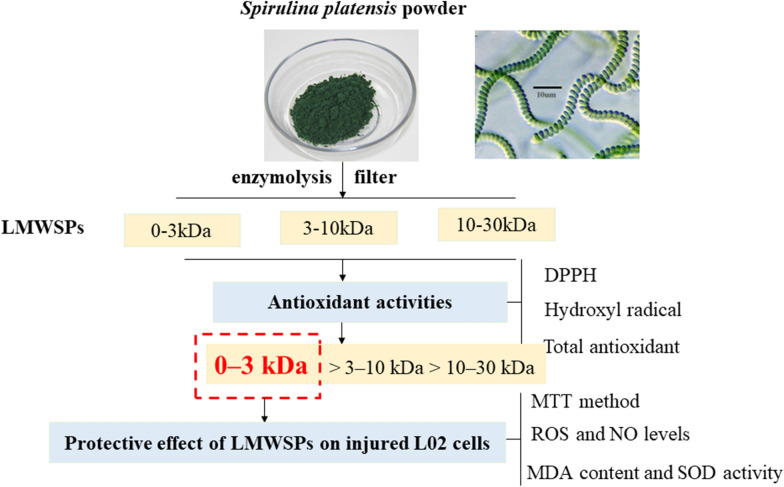

## Introduction

Reactive oxygen species (ROS) are produced by mitochondria during aerobic metabolism and are continuously generated and eliminated in the intracellular compartments of living organisms. This process plays an important causative role in cell signaling in a variety of physiological and pathological processes (Checa and Aran 2020; Ray et al. 2012). ROS imbalance in living organisms can lead to cell necrosis, cell apoptosis or tissue damage through different routes, such as causing lipid peroxidation of polyunsaturated fatty acids, which can lead to changes in macromolecules, especially DNA, thereby leading to apoptosis and necrosis (Valko et al. 2007; Stadtman 2004). Fatty acid oxidation can produce hepatic toxic active oxygen to accelerate oxidative stress response, making fatty liver to transform into hepatitis and liver fibrosis faster (Valko et al. 2006). Currently, people use antioxidants to inhibit oxidation and maintain a balance among the various oxidative processes. However, some synthetic antioxidants used in food products, such as butylated hydroxyanisole (BHA) and butylated hydroxytoluene (BHT), may have potential health risks (Ito et al. 1985). For this reason, peptides have gained popularity as safe natural antioxidants in the food industry and in related fields. Many potent antioxidant protein hydrolysates have been isolated from plants, animals, and microorganisms, and these compounds have exhibited strong antioxidant abilities in model systems. Examples include protein hydrolysates from Melinjo (*Gnetumgnemon*) seeds, spotless smoothhound (*Mustelus griseus*), sweet potato, cod (*Gadusmorhua*), Cornu Bubali (the muscle of yellow stripe trevally; *Selaroides leptolepis*), and corn gluten meal (Supriyadi et al. 2019; Tao et al. 2018; Zhang et al. 2020; Farvin et al. 2016; Hu et al. 2020a, b).

As nutrient-dense foods, low-molecular-weight peptides (LMWPs) have gained popularity because of their advantages over macromolecular peptides. For example, some studies have reported that small peptides from soybean hydrolysate have a higher rate of absorption than large peptides in the intestinal perfusion model of the rat small intestine. Many small-molecule antioxidant peptide hydrolysates isolated from natural materials, such as whey protein hydrolysate peptides, duck skin peptides, gelatin polypeptides of Jellyfish (*Rhopilema esculentum*), chickpea protein hydrolysate (CPH), and soy protein hydrolysates, have been reported to exhibit a high free radical scavenging capacity or cellular antioxidant activity, and several antioxidants have already been incorporated into health foods (Kleekayai et al. 2020; Lee et al. 2013; Zhuang et al. 2010; Xu and Galanopoulos 2019; Tian et al. 2020). In addition, LMWPs have been found to exhibit functional activities, including antimicrobial and anticancer effects (Gan et al. 2020; Bayram et al. 2017).

*Spirulina platensis*, a microscopic blue green alga, is considered healthy due to its high nutritional value and pharmacological activity. The high nutritional value of *Spirulina* peptides from Algeria has been confirmed by rigorous biological and chemical testing (Verdasco-Martín et al. 2019). As an important resource of bioactive peptides, *Spirulina* protein hydrolysates have the ability to inhibit tumors, antibacterial and oxidation-induced damage (Wang and Zhang 2016a, b; Sun et al. 2018; Akpinar and Yumusak 2018) The ACE inhibitory peptide purified from *Spirulina platensis* has exhibited potential preventive and therapeutic effects on hypertension (Lu et al. 2010). In addition, 15 polypeptides from *Spirulina* have been shown to have anti-proliferation activities in five cancer cells (HepG-2, MCF-7, SGC-7901, A549, and HT-29) (Wang and Zhang 2016a, b). In our study, to explore the effect of LMWSPs on liver oxidation and protection, we used LO2 human normal liver cells to establish oxidative stress model. The purpose of this study was to screen *Spirulina platensis* hydrolytic fragments (30, 10, and 3 kDa) with high oxidation resistance by chemical antioxidant method, and to study the antioxidant activity of LMWSPs on H_2_O_2_-induced oxidative damage in L02 human liver cells, exploring the effect of LMWSPs on liver oxidation and protection.

## Materials and methods

### Materials

*Spirulina platensis* powder was provided by the *Spirulina platensis* Research Institute of Beijing Forest University (Beijing, China). Papain (≥ 3 U/mg) was purchased from Aladdin Reagent Company (Shanghai, China). DPPH was purchased from Sigma-Aldrich Co. (St. Louis, MO, USA). A T-AOC kit was bought from the Nanjing Built Biological Engineering Research Institute (Nanjing, China). 3-[4,5-Dimethylthiazol-2-yl]-2,5-diphenyltetrazoliumbromide (MTT) and DCFH-DA were purchased from Sigma-Aldrich Co. (St. Louis, Mo, USA). 3-Amino,4-aminomethyl-2′,7′-difluorescein diacetate (DAFFM-DA) was obtained from Beyotime (Jiangsu, China). The other chemicals were of analytical grade and were commercially available.

Human normal liver cell lines (L02) was purchased at National Infrastructure of Cell Line Resource and approved by Ethics Committee of Peking Union Medical College (0111003). Experiments were implemented in compliance with the Helsinki Declaration.

### Extraction of protein and concentration determination

First, *Spirulina platensis* powder (20 g) was mixed with distilled water (160 mL), frozen using liquid nitrogen, and thawed via the water bath method (25–30 ℃). After repeating these two steps five times, the mixture was subjected to ultrasonication at room temperature and 150 W for 3 min. The sample was centrifuged at 6000 rpm at 4 ℃ for 30 min. The supernatant (protein fraction) was immediately subjected to freeze drying using a vacuum freeze dryer, and the resulting *Spirulina platensis* protein powder was stored at 4 ℃.

### Preparation of *Spirulina platensis* peptides

The protein concentration was estimated to be 56 ± 0.97%. The hydrolysate was obtained by subjecting the *Spirulina platensis* protein powder to enzymolysis. Three compounds, ethylene diamine tetra acetic (EDTA), l-cysteine, and NaCl, were added to 30 mL of 2% *Spirulina* protein liquid, and the final concentrations of the solvents were adjusted to 2.0 mM, 5.0 mM, and 300 mM, respectively. The papain enzymatic hydrolysis conditions included a temperature of 60 ℃ for 9 h at pH 7.0, and the ratio of enzyme and substrate was 1.6%. The resulting hydrolysate was filtered with 30, 10, and 3 kDa MW cutoff ceramic membranes successively in ultrafiltration centrifuge tubes at 6000 rpm at 4 ℃, for 30 min and stored at 4 ℃ after vacuum freeze drying. The antioxidant abilities of the filtered peptides of different molecular weights were individually determined in vitro.

### Quantitation of *Spirulina platensis* peptides

The *Spirulina platensis* peptides (crude extract protein, 0–3 kDa, 3–10 kDa and 10–30 kDa) concentration was detected by bicinchoninic acid (BCA) assay. The experimental operation steps according to the BCA Protein Assay Kit (Yuangye Biotech CO., Shanghai, China). The absorbance was measured at 562 nm using a microplate reader (PerkinElmer, Shanghai, China).

### Antioxidant activity assay

#### DPPH radical scavenging activity assay

Aliquots of 0.5 mL of three different molecular weights of *Spirulina platensis* peptides (0–3 kDa, 3–10 kDa and 10–30 kDa) and Vc in ethanol were added to 2.5 mL 0.1 mM DPPH in 100% ethanol. The mixture was shaken and allowed to stand at room temperature in the dark for 30 min, and the absorbance of the resulting solution was measured at 517 nm (AS). Ethanol was used instead of samples in the control experiment (AC), and the blank was prepared as described above but without DPPH (AB). A lower absorbance represented a higher DPPH scavenging activity. The scavenging activity was calculated as $\left[ {1 - \frac{{{\text{AS}} - {\text{AB}}}}{{{\text{AC}}}}} \right] \times 100\%$.

#### Hydroxyl radical scavenging activity assay

The reaction mixture containing 2 mL of 2.0 mM FeSO_4_, 1 mL of 6 mM salicylate in ethanol, 5 mL of samples, and 2 mL of 6.0 mM H_2_O_2_ was incubated for 15 min at 37 ℃ in a water bath. After incubation, the absorbance of the resulting solution was measured at 510 nm (AS) using a spectrophotometer. The control was prepared in the same manner except that distilled water was used instead of the samples (AC). The abilities to scavenge hydroxyl radicals were calculated as $\frac{{{\text{AC}} - {\text{AS}}}}{{{\text{AC}}}} \times 100\% .$

#### Total antioxidant capacity assay

The total antioxidant capacity was assayed using the T-AOC kit. The absorbance of solution was measured at 517 nm using a spectrophotometer. The total antioxidant capacities were calculated according to T-AOC kit’s manufacturer.

### Determination of cell viability

1 × 10^4^ cells/mL (100 μL per well) L02 were planted into 96-well plates and cultured for 24 h. The cells were maintained in an atmosphere of 5% CO_2_ at 37 ℃ in DMEM that was supplemented with 10% fetal bovine serum and 0.1% antibiotics (penicillin and streptomycin). When the cells were completely adhered to the wall, they were treated with various concentrations of LMWSPs (0–3 kDa) and the oxidative damaging agent, H_2_O_2_ (1000 μg/mL or 2000 μg/mL). After 24 h of incubation, 10 μL of MTT (5 mg/mL) in phosphate buffered saline (PBS) was added to each well, and the plates were placed in the dark. After four hours, the liquids in the upper layer were removed, and 100 μL of DMSO was added to the 96-well plates to dissolve the purple formazan crystals. The L02 cell viability was estimated by reading the absorbance at 492 nm using a microplate reader (PerkinElmer, Shanghai, China). The relative cell viability was defined as the absorbance of treated wells divided by that of the control.

### Intracellular reactive oxygen species (ROS) detection

The level of intracellular ROS was detected using the DCFH-DA assay. 1 × 10^4^ cells/mL (100 μL per well) L02 were planted into 96-well plates and cultured for 24 h. When the cells were completely adhered to the wall, they were treated with various concentrations of LMWSPs (0–3 kDa) and the 2000 μg/mL H_2_O_2_. The L02 cells were washed mildly twice with PBS and incubated in Krebs’ ringer solution containing 10 μM DCFH-DA at 37 ℃ for 30 min. After three additional washes with PBS, the DCF-labeled L02 cells were observed and scanned under a confocal microscope (FV 500, Olympus, Japan). Digital pictures were analyzed using Image-J software, and the results were used to calculate the integrated optical density (IOD), which increases as the level of intracellular ROS rises.

### Intracellular nitric oxide (NO) test

The intracellular nitric oxide level was determined using a fluorescent NO-sensitive dye via the DAFFM DA assay. Shortly after sucking out the cell culture fluid, cells were washed twice with PBS and maintained in the Krebs’ ringer solution supplemented with 10 μM DAFFM DA at 37 ℃ for 20 min. After three additional washes with PBS to remove excess dye, the cells were examined and scanned using a confocal microscope. Digital pictures were processed using Image-J software, and the results were used to calculate the IOD, which increases as the level of intracellular NO rises.

### Analysis of antioxidant activities

Determination of the MDA content and SOD activity are often combined with each other, because the level of SOD activity indirectly reflects the ability of the body to clear oxygen free radicals and MDA indirectly reflects the severity of free radical attack on the cells. The MDA and SOD levels in the supernatants were estimated using MDA and SOD assay kits, respectively, from Jiancheng Bioengineering Institute (Nanjing, China). Briefly, the supernatants were collected, and the absorbance was read at 532 nm using a spectrophotometer, and the MDA and SOD contents were calculated as nmol/mL protein and U/mL, respectively.

### Statistical analysis

Statistical analyses were compared via one-way analysis of variance (ANOVA), all calculations were performed using IBM SPSS statistics 21.

## Results

### Antioxidant activities of the *Spirulina *peptide hydrolysates

**Fig. 1:**
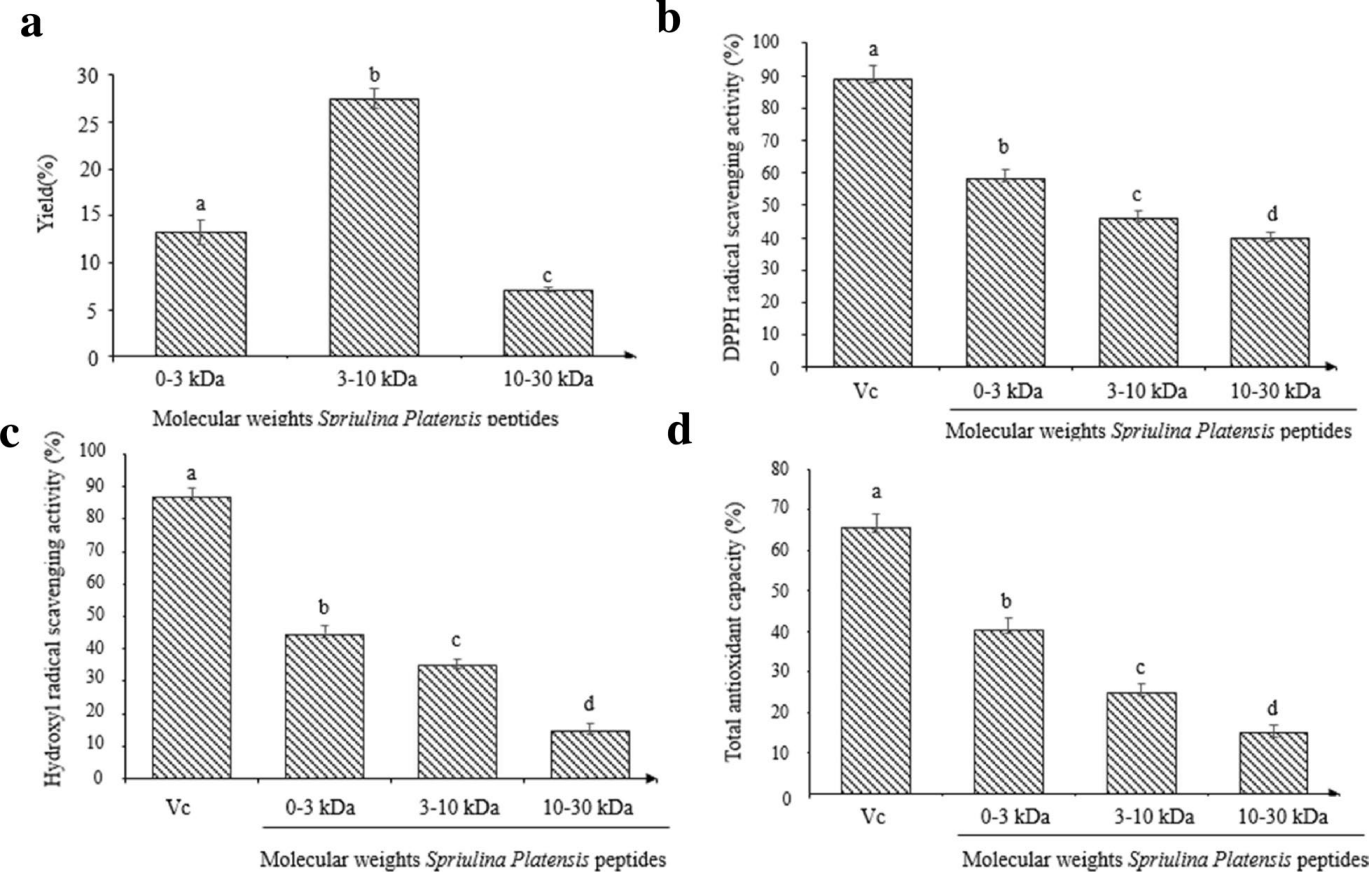
Antioxidant activities of the papain hydrolysates of *Spirulina platensis* peptides. **a** Preparation yield of the three different molecular weights of *Spirulina platensis* peptides. **b** DPPH radical scavenging activity of the three different molecular weights of *Spirulina platensis* peptides. **c** Hydroxyl radical scavenging activity of the three different molecular weights of *Spirulina platensis* peptides. **d** Total antioxidant capacity of the three different molecular weights of *Spirulina platensis* peptides. Vc as positive control. The bar graph represents mean ± SE (*n* = 3)

The antioxidant activities of the peptide hydrolysates of *Spirulina platensis* are shown in Fig. 1. At the concentration of 1.0 mg/mL, the DPPH scavenging activities, OH· scavenging activities, and total antioxidant abilities decreased in the following order: 0–3 kDa > 3–10 kDa > 10–30 kDa. Hydrolysates with molecular weights ranging from 0 to 3 kDa had the highest antioxidant activities (*P* < 0.05), which was lower than those of Vc at the concentration of 0.5 mg/mL.

### Effects of L02 cell pretreatment with H_2_O_2_ and LMWSPs

**Fig. 2 a:**
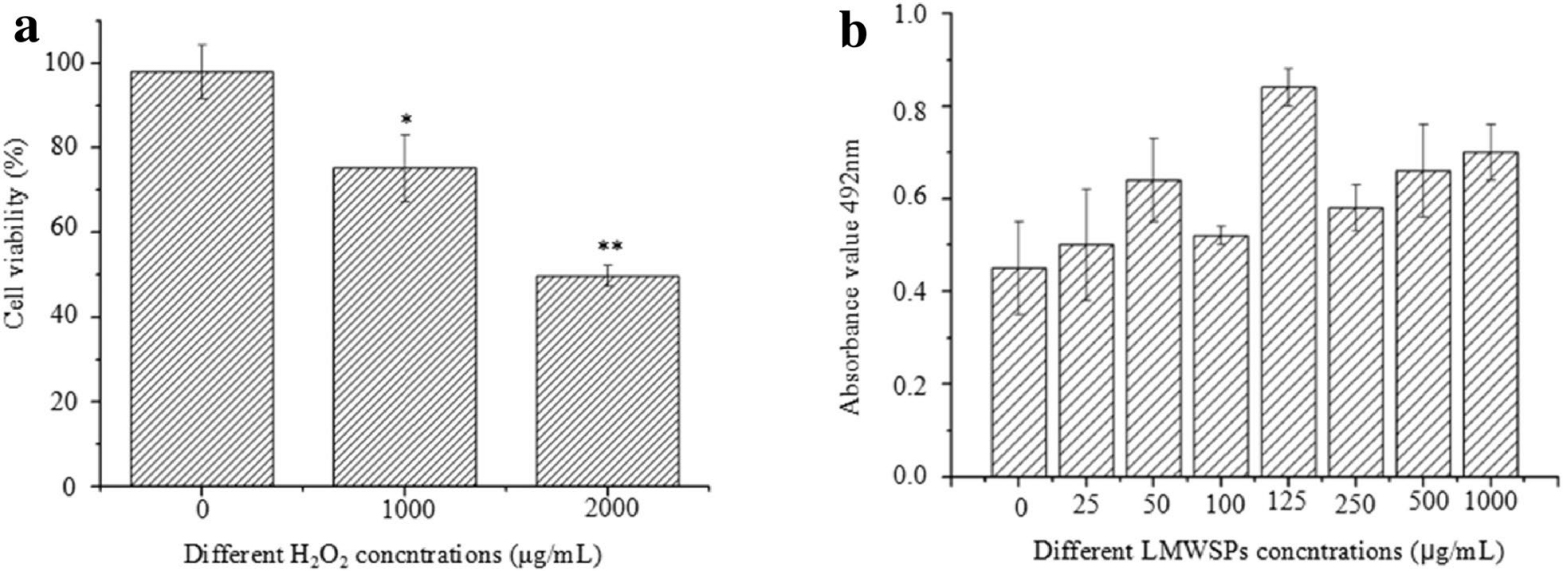
Viability of L02 cell pretreatment with H_2_O_2_ and LMWSPs by the MTT assay. Cells were incubated with the indicated concentrations of H_2_O_2_ for 24 h, and the effects of H_2_O_2_ on the L02 cells were analyzed. **b** Effects of LMWSPs in different concentrations (0–1000 μg/mL) on the L02 cells. The bar graph represents mean ± SE (*n* = 3), *P < 0.05, **P < 0.01 (compared with the blank control)

The viability of the L02 cells after incubation with 1000 μg/mL or 2000 μg/mL of H_2_O_2_ was measured using the MTT method. Toxicity analysis revealed that the extent of oxidative damage increased with H_2_O_2_ concentration. The L02 cells showed significantly decreased cell viability compared with the control group, almost 50% of the cells was inhibited when the H_2_O_2_ concentration was 2000 μg/mL. (Fig. 2a, *P* < 0.01); therefore, we chosed 2000 μg/mL H_2_O_2_ as the appropriate concentration to induce oxidative damage in all further experiments. In addition, 0–3 kDa of *Spirulina* peptides individually at various concentrations (25, 50, 100, 125, 250, 500, and 1000 μg/mL) did not cause any apparent cytotoxic effects. Instead, they promoted cell growth, as assessed using the MTT test (Fig. 2b).

### Effect of LMWSPs on cells impaired by H_2_O_2_ treatment

**Fig. 3:**
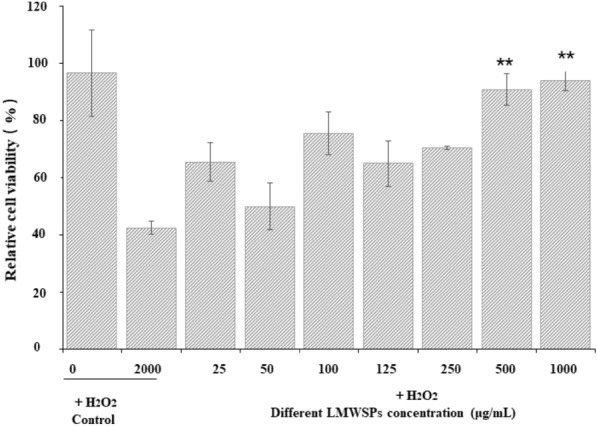
Protective effects of LMWSPs against H_2_O_2_-induced oxidative stress on the L02 cells. Data are expressed as mean ± SE, *n* = 3, **P < 0.01 (compared with H_2_O_2_ control group)

Pretreatments with different concentrations of 0–3 kDa *Spirulina* peptides (25, 50, 100, 125, 250, 500, and 1000 μg/mL) after the addition of 2000 μg/mL H_2_O_2_ for 24 h, the effect of *Spirulina* peptides against H_2_O_2_-induced oxidative stress on the L02 cells was assessed as shown in Fig. 3 The viability of cells treated with various concentrations of 0–3 kDa *Spirulina* peptides was higher than that observed for non-treated cells (2000 μg/mL H_2_O_2_-induced) (*P* < 0.05), In particular, 1000 μg/mL of LMWSPs restored cell viability to near normal levels.

### Effects of LMWSPs on ROS and NO levels in H_2_O_2_-induced cells

**Fig. 4:**
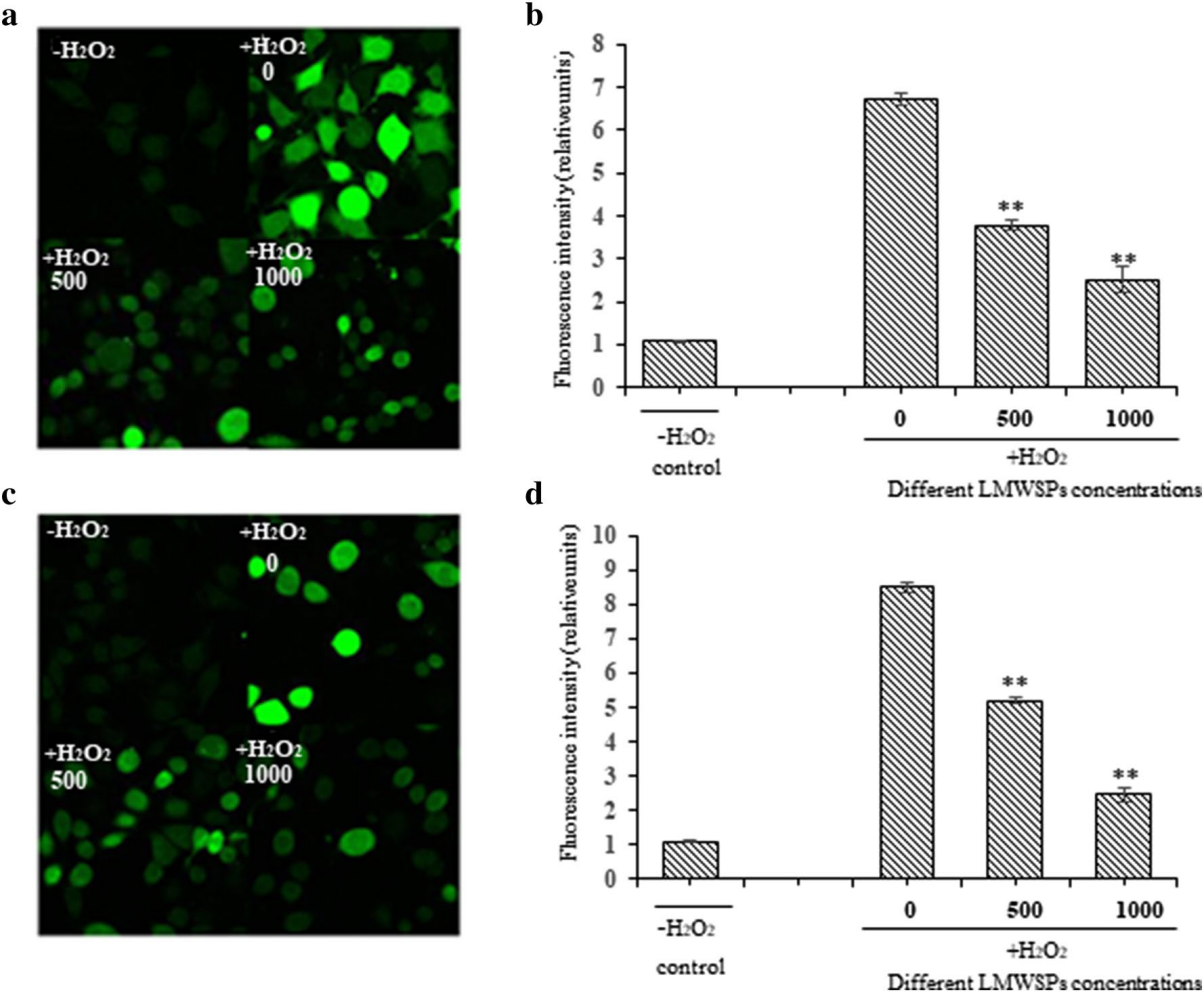
Protective effect of LMWSPs on L02 cells impaired by H_2_O_2_. **a** ROS level was determined by measuring the fluorescence of L02 cells under a confocal microscope. a: Wt; b: 2000 H_2_O_2_ + 0 peptides; c: 2000 H_2_O_2_ + 500 μg/mL peptides; d: 2000 H_2_O_2_ + 1000 μg/mL peptides. **b** Quantification of intracellular ROS production in damaged and normal cells. Data are expressed as mean ± SE, *n* = 3, **P < 0.01 (compared with H_2_O_2_ control group). **c** NO level was determined by measuring the fluorescence of L02 cells under a confocal microscope. a: Wt; b: 2000 H_2_O_2_ + 0 peptides; c: 2000 H_2_O_2_ + 500 μg/mL peptides; d: 2000 H_2_O_2_ + 1000 μg/mL peptides. **d** Quantification of intracellular NO production in damaged and normal cells. Data are expressed as mean ± SE, *n* = 3, **P < 0.01 (compared with H_2_O_2_ control group)

ROS is an important index used to evaluate oxidative stress. The LMWSPs had an inhibitory effect on the abnormal increase of ROS in injured L02 cells. The intracellular ROS level in the L02 cells was approximately 6.23 times greater than that in control cells damaged with 2000 μg/mL H_2_O_2_ (*P* < 0.01). The ROS level in the experimental group that was treated with 500 μg/mL and 1000 μg/mL LMWSPs for 24 h decreased significantly to 46.93% and 36.73% compared with the damaged group, respectively, which was treated with 2000 μg/mL H_2_O_2_ (*P* < 0.01; Fig. 4a, b). The level of intracellular NO is another indicator of oxidative stress. As shown in Fig. 4c, d. Similar to the ROS levels, treatment with 500 μg/mL and 1000 μg/mL of LMWSPs visibly decreased to 60.96% and 28.49% compared with damaged group (*P* < 0.01; Fig. 4c, d).

### Effects of LMWSPs on MDA content and SOD activity in L02 cells

**Fig. 5:**
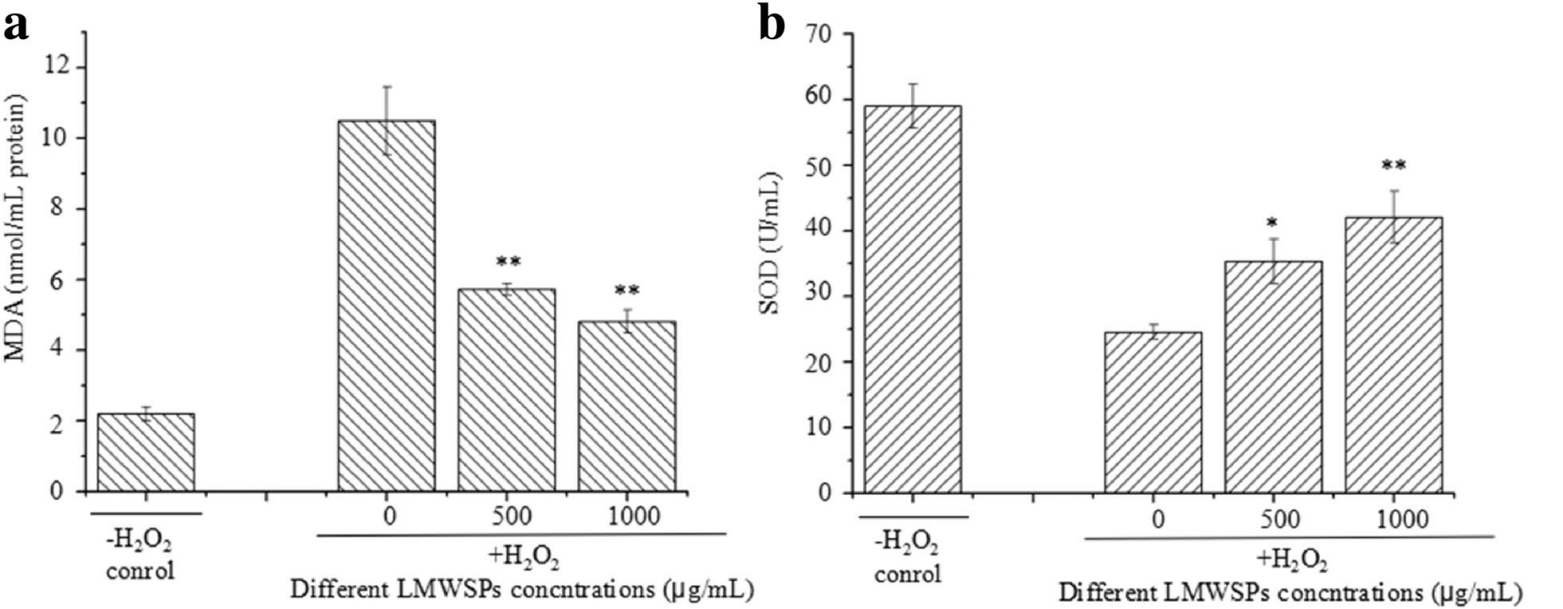
Effect of LMWSPs on the MDA content and SOD activity in H_2_O_2_-treated L02 cells. Cells were pretreated with different concentrations of 0–3 kDa LMWSPs for 24 h before treatment with H_2_O_2_ for 24 h. Each column represents the mean ± SE (*n* = 3). *P < 0.05, **P < 0.01 (compared with the H_2_O_2_ control group)

The MDA activity test showed a clear trend in the L02 cells. The MDA content in the H_2_O_2_-treated L02 cells was 8.27 ± 0.17 nmol/mL, which was approximately 7.59 times than that in the control group (*P* < 0.01; Fig. 5a). As the concentrations of the LMWSPs were increased, the MDA contents decreased gradually. 500 and 1000 μg/mL of LMWSPs effectively inhibited the increase of MDA contents by 34.7% and 71.5% (*P* < 0.01; Fig. 5a), respectively, compared with the H_2_O_2_-treated group. Compared with the SOD activity in the control group, the SOD activity of the H_2_O_2_-treated group decreased significantly by 58.37% (*P* < 0.01; Fig. 5b). Compared with the H_2_O_2_-treated group, the SOD activity of the group treated with 500 and 1000 μg/mL LMWSPs was 35.37 ± 3.41 U/mL and 42.10 ± 3.88 U/mL, which was a significant increase of 44.01% and 71.42% (*P* < 0.05; *P* < 0.01, Fig. 5b).

## Discussion

The ROS index, which includes hydrogen peroxide (H_2_O_2_), Superoxide radical (O^2^*^−^) and hydroxyl radical (*OH), etc., is an important index for evaluating oxidative stress (Wang and Jiao 2000). Free radical imbalance in an organism is responsible for many pathological conditions, such as Alzheimer’s disease, atherosclerosis, neurodegenerative disease and coronary heart disease (Moneim 2015; Singh et al. 2019; Georgieva et al. 2017; Stemoukhov et al. 2001). Free radicals are quenched by antioxidants during cell metabolism in normal cells, after which they can be effectively eliminated by the antioxidant defense system, which involves several enzymes (Nimse and Pal 2015). In this study, we used H_2_O_2_-induced L02 cells injury to examine the effects of LMWSPs on oxidative stress-induced retinal damage in L02 cells. We observed that H_2_O_2_ treatment caused a significant decrease in L02 cells viability. However, LMWSPs treatment increased the viability in human L02 cells expose to H_2_O_2_. These results suggest that the protective effect of LMWSPs oxidative stress in L02 cells. This observation was consistent with the results of previous studies (Xia et al. 2017; Jian et al. 2017). We detected the level of intracellular ROS and NO to explore the antioxidant activity of LMWSPs. Our results suggested that LMWSPs could alleviate the intracellular oxidation process. In addition, treatment with 1000 μg/mL of LMWSPs effectively prevented the increase in MDA content and inhibited the reduction of SOD activity in the H_2_O_2_-induced L02 cells, and it alleviated the susceptibility of the cells to secondary oxidative damage by H_2_O_2_.

Several studies have reported that the bioactivity of peptide mixtures is largely affected by their molecular sizes and that the low-molecular-weight fractions exhibit more potent antioxidant activities, which may be due to the presence of hydrophobic amino acids in low-molecular-weight peptide. (Dong et al. 2017). For example, low-molecular-weight whey hydrolysate peptides (0.1–2.8 kDa) have been found to be highly antioxidative and can protect lung fibroblast MRC-5 cells against H_2_O_2_-induced oxidative damage (Kong et al. 2012). One previous report also showed that low-molecular peptides (941.43 Da) from duck skin by-products have high radical scavenging activities and protect the liver against oxidative damage. In the previous studies, the 0–3 kDa of Monkfish muscle proteins hydrolysate (MPTH-I), Blood plasma hydrolysate (BPH4) and peptides derived from biodegradation of chicken feather (PBCF) showed the highest antioxidant activities than other molecular weights, corroborating our result that LMWSPs (0–3 kDa) have highest antioxidant activities (Hu et al. 2020a, b; Cheng et al. 2016; Alahyaribeika et al. 2021). Table 1 shows that the difference of antioxidant activities between peptides (0–3 kDa) derived from four different species. The DPPH radical scavenging activity of LMWSPs (0–3 kDa) was 58.3% ± 2.89% at a concentration of 1.0 mg/mL, which was stronger than that of MPTH-I and PBCF. In addition, the results of hydroxyl radical scavenging activity shows that LMWSPs (0–3 kDa) has higher antioxidant activity than MPTH-I.

**Table 1:** Comparison of antioxidant activities between peptides (0–3 kDa) of four different species

Fractions	Molecular weight	Concentration (mg/mL)	DPPH radical scavenging activity	Hydroxyl radical scavenging activity	References
LMWSPS	0–3 kDa	1.0	58.3% ± 2.89%	44.5% ± 2.8%	This study
MPTH-I		5.0	65.52% ± 3.86%	58.78% ± 3.05%	Hu et al. (2020a, b)
BPH4		1.0	69.9% ± 3.5%	ND	Cheng et al. (2016)
BCFP		5.03	50%	ND	Alahyaribeika et al. (2021)

*LMWSPs* low-molecular-weight Spirulina peptides, *MPTH-I* Monkfish muscle proteins hydrolysate, *BPH4* blood plasma hydrolysate, *PBCF* peptides derived from biodegradation of chicken feather, *Note* no detected

The safety of *Spirulina* peptides in health foods has attracted much attention, and most studies have concentrated on the cell apoptosis-triggering and anticancer effects of the LMWSPs (Wang and Zhang 2015). Our results reveal that different concentrations of *Spirulina* peptides promote cell growth and are non-toxic to a normal human cells line (L02). Similar results have been obtained with mulberry leaf low-molecular-weight peptides, which have a beneficial effect on silkworm growth and development (Jha et al. 2014). In addition, LMWSPs may be involved in regulating cell proliferation-related gene expression. Our results provide a theoretical foundation for using LMWSPs as a safe and functional food.

## Conclusions

Our study demonstrates that LMWSPs (0–3 kDa) have stronger antioxidant properties than large-molecular-weight *Spirulina* peptides (3–10, 10–30 kDa). Furthermore, LMWSPs have a protective effect on L02 cells by reducing oxidative stress and improving cell viability without any toxic influence on normal liver cells. Our findings provide the basis for further research on functional food or theoretical medicine to alleviate oxidative stress in human liver cells from oxidative injury.

## Data Availability

All data generated or analyzed during this study are included in this article.

## Abbreviations

LMWSPs: Low-molecular-weight *Spirulina* peptides
DPPH: 1,1-Diphenyl-2-picrylhydrazyl
·OH: Hydroxyl radical
MPTH-I: Monkfish muscle proteins hydrolysate
BPH4: Blood plasma hydrolysate
PBCF: Peptides derived from biodegradation of chicken feather
Vc: Vitamin C
DCFH-DA: 2′,7′-Dichlorofluorescein diacetate
ROS: Reactive oxygen species
BHA: Butylated hydroxyanisole
BHT: Butylated hydroxytoluene
MTT: 3-[4,5-Dimethylthiazol-2-yl]-2,5-diphenyltetrazo-liumbromide
DAFFM-DA: 3-Amino,4-aminomethyl-2′,7′-difluorescein diacetate
L02: Human normal liver cell lines
EDTA: Ethylene diamine tetra acetic
PBS: Phosphate buffered saline
ROS: Intracellular reactive oxygen species
IOD: Integrated optical density
NO: Intracellular nitric oxide
GSH: Glutathione

## References

[CR1] Akpinar M, Yumusak N (2018). Production of bioactive proteins and peptides from the diatom *Nitzschia laevis* and comparison of their in vitro antioxidant activities with those from *Spirulina platensis* and *Chlorella vulgaris*. Int J Food Sci Technol.

[CR2] Alahyaribeika S, Sharifia SD, Tabandehb F, Honarbakhsha S, Ghazanfaria S (2021). Stability and cytotoxicity of DPPH inhibitory peptides derived from biodegradation of chicken feather. Protein Expr Purif.

[CR3] Bayram B, Ozgur A, Tutar L, Tutar Y (2017). Tumor targeting of polymeric nanoparticles conjugated with peptides, saccharides, and small molecules for anticancer drugs. Curr Pharm Des.

[CR4] Checa J, Aran JM (2020). Reactive oxygen species: drivers of physiological and pathological processes. J Inflamm Res.

[CR5] Cheng FY, Lai IC, Lin LC, Sakata R (2016). The in vitro antioxidant properties of alcalase hydrolysate prepared from silkie fowl (*Gallus gallus*) blood protein. Anim Sci J.

[CR6] Dong ZY, Tian G, Xu ZG, Li MY, Xu M, Zhao YJ, Ren H (2017). Antioxidant activities of peptide fractions derived from freshwater mussel protein using ultrasound-assisted enzymatic hydrolysis. Czech J Food Sci.

[CR7] Farvin KHS, Andersen LL, Otte J (2016). Antioxidant activity of cod (*Gadus morhua*) protein hydrolysates: fractionation and characterisation of peptide fractions. Food Chem.

[CR8] Gan BH, Cai XG, Javor S, Köhler T, Reymond JL (2020). Synergistic effect of propidium iodide and small molecule antibiotics with the antimicrobial peptide dendrimer G3KL against gram-negative bacteria. Molecules.

[CR9] Georgieva E, Ivanov D, Zhelev Z, Bakalov R, Gulubov M, Aoki I (2017). Mitochondrial dysfunction and redox imbalance as a diagnostic marker of “free radical diseases”. Anticancer Res.

[CR10] Hu RJ, Dunmire KM, Truelock CN, Paulk CB, Aldrich G, Li YH (2020). Antioxidant performances of corn gluten meal and DDGS protein hydrolysates in food, pet food, and feed systems. J Agric Food Res.

[CR11] Hu XM, Wang YM, Zhao YQ (2020). Antioxidant peptides from the protein hydrolysate of Monkfish (*Lophius litulon*) muscle: purification, identification, and cytoprotective function on HepG2 cells damage by H_2_O_2_. Mar Drugs.

[CR12] Ito N, Fukushima S, Tsuda H (1985). Carcinogenicity and modification of the carcinogenic response by BHA, BHT, and other antioxidants. Crit Rev Toxicol.

[CR13] Jha S, Mandal P, Bhattacharyya P, Ghosh A (2014). Free-radical scavenging properties of low molecular weight peptide(s) isolated from S1 cultivar of mulberry leaves and their impact on *Bombyx mori* (L.) (Bombycidae). J Anim Sci Biotechnol.

[CR14] Jian WJ, Tu LY, Wu LL, Xiong HJ, Pang J, Sun YM (2017). Physicochemical properties and cellular protection against oxidation of degraded Konjac glucomannan prepared by γ-irradiation. Food Chem.

[CR15] Kleekayai T, Gouic AVL, Deracinois B, Cudennec B, FitzGerald RJ (2020). In vitro characterisation of the antioxidative properties of whey protein hydrolysates generated under pH- and non pH-controlled conditions. Foods.

[CR16] Kong BH, Peng XY, Xiong YLL, Zhao XH (2012). Protection of lung fibroblast MRC-5 cells against hydrogen peroxide-induced oxidative damage by 0.1–2.8 kDa antioxidative peptides isolated from whey protein hydrolysate. Food Chem.

[CR18] Lee SJ, Cheong SH, Kim YS, Hwang JW, Kwon HJ, Kang SH, Moon SH, Jeon BT, Park PJ (2013). Antioxidant activity of a novel synthetic hexa-peptide derived from an enzymatic hydrolysate of duck skin by-products. Food Chem Toxicol.

[CR19] Lu J, Ren DF, Xue YL, Sawano Y, Miyakawa T, Tanokura M (2010). Isolation of an antihypertensive peptide from alcalase digest of *Spirulina platensis*. J Agric Food Chem.

[CR20] Moneim AEA (2015). Oxidant/antioxidant imbalance and the risk of Alzheimer’s disease. Curr Alzheimer Res.

[CR21] Nimse SB, Pal D (2015). Free radical, natural antioxidants, and their reaction mechanisms. RSC Adv.

[CR22] Ray PD, Huang BW, Tsuji Y (2012). Reactive oxygen species (ROS) homeostasis and redox regulation in cellular signaling. Cell Signal.

[CR23] Singh AJ, Kukreti R, Saso L, Kukreti S (2019). Oxidative stress: a key mdulator in neurodegenerative diseases. Molecules.

[CR24] Stadtman ER (2004). Role of oxidant species in aging. Curr Med Chem.

[CR25] Stemoukhov AA (2001). Histamine-dependent changes in free radical processes during coronary heart disease. Bull Exp Biol Med.

[CR26] Sun YJ, Chang R, Li PY, Li BS (2018). Isolation and characterization of an antibacterial peptide from protein hydrolysates of *Spirulina platensis*. Eur Food Res Technol.

[CR27] Supriyadi A, Arum LS, Nugraha AS, Ratnadewi AAI, Siswoyo TA (2019). Revealing antioxidant and antidiabetic potency of Melinjo (*Gnetum Gnemon*) seed protein hydrolysate at different stages of seed maturation. Curr Res Nutr Food Sci.

[CR28] Tao J, Zhao YQ, Wang B, Chi CF (2018). Bioactive peptides from cartilage protein hydrolysate of spotless smoothhound and their antioxidant activity in vitro. Mar Drugs.

[CR29] Tian R, Feng J, Huang G, Tian B, Zhang Y, Jiang LZ, Sui XN (2020). Ultrasound driven conformational and physicochemical changes of soy protein hydrolysates. Ultrason Sonochem.

[CR30] Valko M, Rhodes CJ, Moncol J, Izakovic M, Mazur M (2006). Free radicals, metals and antioxidants in oxidative stress-induced cancer. Chem Biol Interact.

[CR31] Valko M, Leibfritz D, Moncol J, Cronin MT, Mazur M, Telser J (2007). Free radicals and antioxidants in normal physiological functions and human disease. Int J Biochem Cell Biol.

[CR32] Verdasco-Martín C, Echevarrieta L, Otero C (2019). Advantageous preparation of digested proteic extracts from *Spirulina platensis* biomass. Catalysts.

[CR33] Wang SY, Jiao HJ (2000). Scavenging capacity of berry crops on superoxide radicals, hydrogen peroxide, hydroxyl radicals, and singlet oxygen. J Argic Food Chem.

[CR34] Wang Z, Zhang X (2015). Inhibitory effects of small molecular peptides from *Spirulina* (*Arthrospira*) *platensis* on cancer cell growth. Food Funct.

[CR35] Wang ZJ, Zhang XW (2016). Characterization and antitumor activity of protein hydrolysates from *Arthrospira platensis* (*Spirulina platensis*) using two-step hydrolysis. J Appl Phycol.

[CR36] Wang ZJ, Zhang XW (2016). Inhibitory effects of small molecular peptides from *Spirulina* (*Arthrospira*) *platensis* on cancer cell growth. Food Funct.

[CR37] Xia T, Yao J, Zhang J, Zheng Y, Song J, Wang M (2017). Protective effects of Shanxi aged vinegar against hydrogen peroxide-induced oxidative damage in LO2 cells through Nrf2-mediated antioxidant responses. RSC Adv.

[CR38] Xu Y, Galanopoulos M (2019). Effect of enzymatic hydrolysis using endo-and exo-proteases on secondary structure, functional, and antioxidant properties of chickpea protein hydrolysates. J Food Meas Charact.

[CR40] Zhuang YL, Sun LP, Zhao X, Hou H, Li BF (2010). Investigation of gelatin polypeptides of jellyfish (Rhopilema esculentum) for their antioxidantactivity in vitro. Food Technol Biotech.

[CR39] Zhang M, Huang TS, Mu TH (2020). Production and characterisation of antioxidant peptides from sweet potato protein by enzymatic hydrolysis with radio frequency pretreatment. Int J Food Sci Technol.

